# Diagnosing Ceiling Effects and Unstable Nonlinearity in Short Ordinal Scales: A TIMSS 2023 Application

**DOI:** 10.3390/bs16091485

**Published:** 2026-08-25

**Authors:** Georgios Sideridis, Mohammed Alghamdi

**Affiliations:** 1 Biostatistics and Research Design (BARD) Center, Boston Children’s Hospital, Harvard Medical School, 300 Longwood Avenue, Boston, MA 02115, USA; 2 Department of Self-Development Skills, King Saud University, Riyadh 12372-2308, Saudi Arabia; mhalghamdi@ksu.edu.sa

**Keywords:** ordinal measurement, ceiling effects, local item dependence, functional form, graded response model, TIMSS 2023

## Abstract

Short ordinal self-report scales are widely used to study children’s digital lives, yet their measurement properties can distort conclusions about nonlinear relationships. We introduced an integrated diagnostic workflow for such scales—covering range, structure, reliability, method variance and functional form—and applied it to the TIMSS 2023 Digital Self-Efficacy scale across all 63 Grade 4 and 47 Grade 8 education-system and benchmarking samples (source database N = 719,881 children; 630,461 with complete seven-item measurement data). The scale was endpoint-concentrated, markedly at Grade 8, losing 83% and 95% of its test information between the mean and two standard deviations above it; an exact marginal calculation from a testlet model gave 80% and 89%. The apparent multidimensionality was better represented as localized covariance among three similarly worded items than as a separable second dimension, and omega hierarchical of 0.79 and 0.83 supported using the total score. In a factorial simulation evaluating the population projection coefficient on the analysis scale, endpoint concentration raised rejection of no curvature from 5.4% to 16.7% with raw summed scores, while latent scoring returned it to 5.5% and raised power from 71% to 88%, whether the item parameters were known or, in a smaller supporting condition, estimated in the analysis sample. Applied to cybervictimization, the quadratic association was attenuated but did not reverse sign once covariates were matched, and its prediction interval included zero. The range and reliability diagnostics behaved similarly on a second scale from the same assessment; broader applicability of the full workflow is proposed on theoretical grounds rather than established here.

## 1. Introduction

Short ordinal self-report scales underpin much of the empirical research on children and digital technology. Four to eight items with three to five ordered categories are used to measure confidence, attitudes, skills and well-being, and the resulting composites are then entered into models that ask increasingly refined questions—whether an association is moderated, whether it differs across countries, and, frequently, whether it is nonlinear. Nonlinear questions are especially attractive when theory supplies competing expectations, because a curvilinear term appears to test both at once.

Such scales have properties that make nonlinear inference fragile. When many respondents select the top category on every item, the scale stops distinguishing among them, and any relationship involving the upper range is compressed regardless of its latent form (Bond & Lang, 2013; Rhemtulla et al., 2012). When a subset of items shares wording or a task format, their residual covariance can mimic a second dimension, a long-recognized threat in test construction (Christensen et al., 2017; Yen, 1993), and a composite that aggregates heterogeneous content can express that heterogeneity as apparent curvature (Marsh et al., 2020; Reise et al., 2013). When the predictor and outcome share a response format, person-level endorsement tendencies contribute to both (Baumgartner & Steenkamp, 2001; Weijters et al., 2010). Finally, conclusions about functional form drawn from a squared term are not invariant to the scaling of the outcome or to the estimator used (Ganzach, 1997; Simonsohn, 2018).

These threats are individually well documented and are usually addressed, if at all, one at a time. What is missing is an integrated procedure that an applied researcher can run before interpreting a nonlinear result, together with evidence about which threats actually matter and under what conditions. This article supplies both. We set out a five-stage diagnostic workflow, applied it to a newly released international scale across 110 education-system and benchmarking samples, and used a factorial simulation to establish when the diagnosed conditions distort inference and whether the workflow detects them. We adopt an explicit interpretive standard for what it means for a threat to “matter,” though we note it was an analytic convention rather than a preregistered criterion: a threat matters when it changes the Type I error rate of a routine test for curvature by a practically consequential margin—as a rule of thumb, roughly a doubling of the nominal 5% rate—or produces a comparable loss of power. We state it here so that the simulation results can be read against a declared benchmark.

Our application is the Trends in International Mathematics and Science Study (TIMSS) 2023 Digital Self-Efficacy scale, seven four-category items asking children how confident they are performing digital tasks. The scale is a useful test case for three reasons. It is new, so a large amount of secondary literature is likely to be built upon it. It is administered to children at two grades in more than sixty systems, so measurement properties can be estimated separately in many independent samples rather than assumed. Finally, it arrives alongside the first TIMSS cyberbullying items, creating an obvious substantive analysis—whether digital confidence protects children from online harm (Livingstone & Smith, 2014) or accompanies greater exposure to it (Livingstone & Helsper, 2010). The protective account predicts that greater confidence accompanies lower victimization (a negative slope); the exposure account predicts that greater confidence accompanies more online activity and hence more exposure and victimization (a positive slope). Because the two predict opposite linear effects, a researcher entertaining both is led to a quadratic: a U-shaped curve (positive quadratic) would place minimum risk at an intermediate, “optimal” level of confidence, whereas an inverted U would place maximum risk there. The scale therefore has exactly the structure our workflow is designed to scrutinize.

Recent work has examined the cross-national comparability of this scale: ALMamari (2026) analyzed Grade 8 data from 44 systems using multiple-group confirmatory factor analysis and alignment optimization, reporting configural invariance, failure of full scalar invariance, and relatively strong approximate invariance. We extended this work by evaluating endpoint concentration and conditional information, dimensional structure under categorical estimation, common endorsement-format variance, and the sensitivity of nonlinear secondary analyses to these measurement properties, across both grades. Invariance testing asks whether a scale measures comparably across groups; the workflow asks how well it measures at all, where in its range it measures well, and which inferences it will support.

## 2. A Diagnostic Workflow for Short Ordinal Scales

Table 1 sets out the workflow. Each stage names a potential failure, the analysis that detects it, and the inferential consequence if it goes undetected. The stages are ordered so that earlier results condition the interpretation of later ones: endpoint concentration determines where on the latent continuum estimates are informative; structure determines whether a total score is defensible; reliability quantifies how much of that score is general rather than specific; method variance bounds how much of any association is attributable to shared format; and functional-form checks establish whether a nonlinear conclusion survives ordinary analytic choices. These five stages trace the path from item responses to a substantive inference—range and structure concern the measurement model, reliability the score, method variance the predictor, and functional form the association—so together they cover the points at which a nonlinear conclusion can fail for a single scale in a single sample. Threats that concern comparison across groups, most importantly measurement invariance, lie deliberately outside this within-scale workflow: invariance asks whether a scale measures the same construct across populations, a complementary question addressed for this scale by ALMamari (2026), and we recommend it as an adjunct rather than a sixth stage. In practice a researcher runs the stages in this order and, at each stage, follows the corresponding entry in the final column of Table 1: the diagnostic result dictates the next analytical decision—where to confine inferences, whether to rescore, whether to retain the total score, how to bound an association, and whether a curvilinear term may be interpreted at all.

The workflow has a minimal diagnostic core—the range, structure and reliability stages—that has modest statistical demands and can be executed with standard ordinal psychometric tools; the method-variance and functional-form stages, and the sensitivity analyses used in this application (bifactor information, cross-validated factor comparison, jackknife meta-analysis, and latent interaction models), are more involved. The range, structure and reliability stages require only the item responses; the method-variance stage additionally requires auxiliary items sharing the response format, and the functional-form stage requires the covariates, sampling weights and cluster identifiers used in the substantive analysis. The diagnostics are descriptive rather than inferential, but we note that using them to choose a downstream specification is a form of model selection and can affect the properties of subsequent tests. What the workflow provides is not a test of whether a substantive hypothesis is true, but a statement of which hypotheses the measure is capable of adjudicating.

## 3. Materials and Methods

### 3.1. Data

We analyzed the TIMSS 2023 international student background files (von Davier et al., 2024b): all 63 Grade 4 systems (source database N = 395,961) and 47 Grade 8 systems (N = 323,920), including benchmarking participants. These are the source samples; the complete seven-item measurement sample comprised 344,715 Grade 4 and 285,746 Grade 8 students (630,461 in total), and the cybervictimization control-specification analyses retained 347,489 and 274,119 students. Sampling, weighting and scaling procedures are documented in the technical report (von Davier et al., 2024a); we followed standard recommendations for secondary analysis of international assessment data (Rutkowski et al., 2010). Per-system source and analytic sample sizes, full system names, national/benchmark status, and every diagnostic appear in Supplementary Table S1.

Denominators differed across analyses, and each rule is stated below. Measurement analyses required only the seven items and retained all systems. The sequence of cybervictimization control specifications additionally required the online-use and endorsement blocks and retained 63 and 45 systems. Held-out functional-form analyses required at least 500 complete cases and at least 20 sampled schools, retaining 63 Grade 4 and 45 Grade 8 samples. Measurement analyses used the seven raw items; the cybervictimization analysis used the released item response theory (IRT) scale score.

### 3.2. Software, Weights, Missing Data and Identification

All analyses were conducted in Python 3.10 with NumPy 2.1, SciPy 1.15 and pandas 2.2; the latent variable models reported in Section 4.3 were estimated in Mplus 9. Equivalent graded response and ordinal factor models can be estimated with the mirt package in R 4.5.3 (Chalmers, 2012), and we provided our implementations so that either route can be used. Because the psychometric estimates come from our own implementations, we validated them against the independent girth package (Sanchez, 2021); agreement was close for discriminations, thresholds and information loss (Supplementary Materials). Graded response and factor models were estimated on unweighted item responses within the system, because sampling weights in TIMSS are constructed for population estimation rather than for item-parameter estimation and are approximately constant within the classroom clusters that generate them; total student weights were used for all descriptive statistics, standardization, and the cybervictimization models, and a stratified weighted-versus-unweighted comparison confirmed that weighting changed item parameters only modestly (Supplementary Materials). Cases with any missing response among the seven items were excluded listwise from the measurement models; listwise deletion for the seven self-efficacy items retained a median of 90.4% of the source sample (range 70.5% to 97.3%); per-system retention relative to the source sample is given in Table S1.

Graded response models were identified conventionally (latent mean and variance fixed; ordered thresholds) and estimated by an expectation–maximization algorithm with 25-point Gauss–Hermite quadrature; factor models were estimated from polychoric correlation matrices by unweighted least squares. For tractability, large samples were subsampled for item-parameter estimation (at most 4000 students for the unidimensional and 1500 for the testlet models; fixed seeds, recorded as n_fit in Tables S1 and S2). The estimation ceiling was set to 200 iterations; under the 5 × 10^−4^ tolerance all 110 unidimensional models converged (median 34 iterations), and per-system convergence and boundary status is tabulated in the Supplementary Materials. Item thresholds were bounded at ±8: widening from ±5 reduced the number of boundary-affected models from 45 to 11 (all at Grade 8) and left the information-loss medians unchanged (83% and 95%). These residual bounds were immaterial: refitting the 11 systems at a wider ±12 limit left their information-loss median identical (97.2%) with none still at the bound, and excluding them altogether moved the Grade 8 grade-level median only from 94.6% to 92.9%. A single system (Hong Kong SAR) retained a discrimination at the upper limit; a wider-bound refit placed it at 3.70 with no material change, and a census of all 110 systems found no other discrimination within 0.5 of the limit. A stratified sensitivity check across 18 systems spanning the full size range confirmed that neither the 4000/1500-case subsampling nor the use of sampling weights was consequential: discriminations shifted by at most 0.18 and the information loss by at most 2.6 percentage points (median 0.4).

### 3.3. Range: Endpoint Concentration and Conditional Information

We computed the percentage of children endorsing the maximum category on all seven items, and fitted a graded response model (Samejima, 1969) within each system, deriving test information and the conditional standard error across the latent continuum. Because the structural analyses indicated local dependence among the first three items, and because the standard graded response model assumes local independence and can therefore overstate information, we additionally fitted a bifactor (testlet) graded response model in which items 1–3 load on a general factor and on a specific factor, using two-dimensional quadrature, and computed information about the general factor marginalizing over the specific factor. Both sets of estimates are reported.

### 3.4. Structure, Reliability and Method Variance

Four confirmatory models were estimated from polychoric correlations (Flora & Curran, 2004): one factor; correlated two factors separating items 1–3 from items 4–7; one factor with correlated residuals among items 1–3; and a bifactor model with a specific factor on items 1–3. Models were compared by the standardized root mean square residual, both in sample and—because the residual-correlation and bifactor specifications are more flexible than the two-factor specification—under cross-validation, in which parameters estimated on a random half of each system were evaluated against the polychoric matrix of the held-out half, averaged over both directions. We computed ordinal omega for the total score, omega for each item subset, and omega hierarchical from the bifactor solution (Flora, 2020; Green & Yang, 2009).

A WLSMV/ULSMV analysis of the 220 per-system model fits would require the asymptotic covariance matrix of the polychoric correlations for each fit; we estimated the models in Python by unweighted least squares rather than running 220 confirmatory models in Mplus (which we reserved for the single-system latent interaction), so mean- and variance-adjusted χ^2^ statistics and nested difference tests are not reported. As a descriptive complement to the residual-based and cross-validated comparison, we report population fit indices computed from the ULS discrepancy (RMSEA, CFI, TLI), which are descriptive, ULS-based approximations rather than WLSMV test statistics. We also note that the per-system counts reported throughout (for example, the number of systems in which one model fits at least as well as another) are descriptive summaries of the cross-system distribution of estimates, not families of inferential tests, so no multiple-comparison correction is applied. Our conclusions concern which of four structures reproduces the item correlations most closely out of sample, and cross-validated residual comparison answers that question while penalizing complexity.

Shared response-format variance was estimated two ways: an external endorsement index, the standardized person mean across the environmental-attitude block, which shares the agreement format but not the content of the self-efficacy items and excludes all outcomes; and a common endorsement-format factor in a random-intercept item-factor model with unit loadings on all agreement-format items, orthogonal to the substantive factors (Maydeu-Olivares & Coffman, 2006). We did not label the latter acquiescence: it is consistent with acquiescent responding but equally with general positive self-perception, shared wording, or substantive covariance among constructs measured in that format, a pattern familiar in international assessment through the attitude–achievement paradox (Van de Gaer et al., 2012). The latent models were estimated in the largest participating system at each grade, the United Arab Emirates (analytic n = 32,329 at Grade 4 and 30,136 at Grade 8, after listwise deletion of cases with missing covariates), with a latent quadratic formed by the Latent Moderated Structural equations method (Klein & Moosbrugger, 2000). Because the latent product term requires maximum likelihood, the four-category indicators were treated as continuous under robust maximum likelihood (a limitation noted below), with a Taylor-series complex-sampling correction using total student weights; the random-intercept factor loaded on the self-efficacy and school-belonging indicators only. Estimation and design-correction details are in the Supplementary Materials.

### 3.5. Variables and Models Used in the Applied Analyses

**Figure 1:**
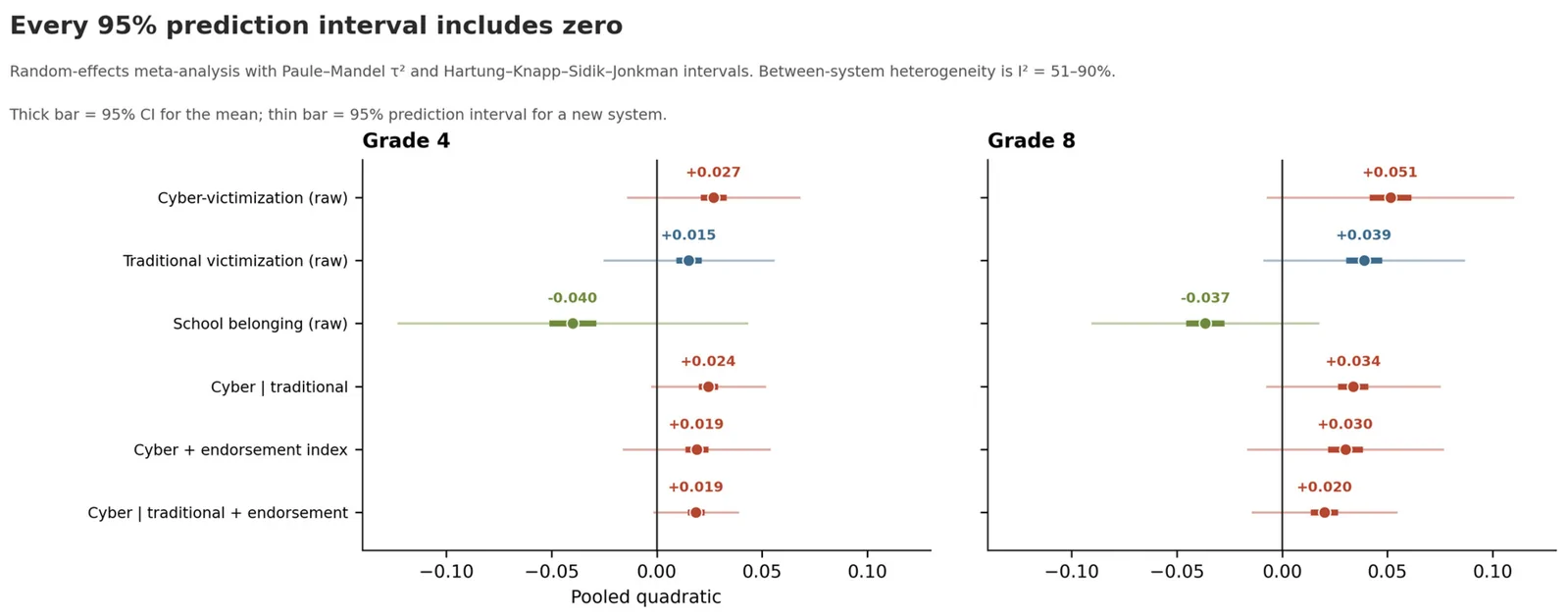
Confidence and prediction intervals for the pooled quadratic. Thick bars are 95% Hartung–Knapp confidence intervals for the pooled mean; thin bars are 95% prediction intervals for a newly sampled education system. Every prediction interval includes zero, reflecting substantial between-system heterogeneity.

**Figure 2:**
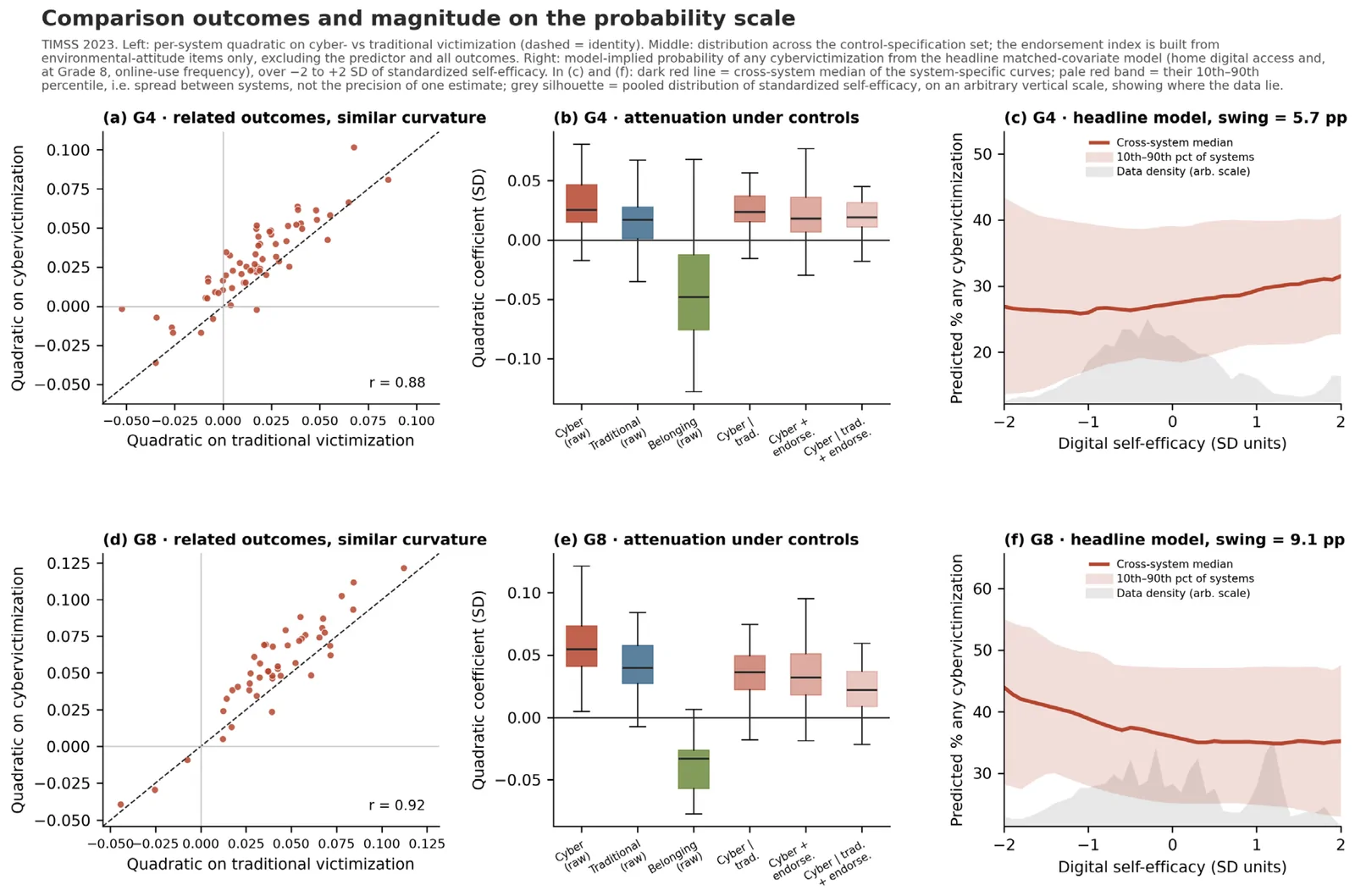
Comparison outcomes and magnitude on the probability scale. Panels (**a**,**d**) plot the per-system quadratic on cybervictimization against that on traditional victimization (dashed line = identity); panels (**b**,**e**) show the cross-system distribution across the control specifications; panels (**c**,**f**) give the model-implied probability of any cybervictimization under the headline matched-covariate model (home digital access and, at Grade 8, online-use frequency), as the cross-system median with the 10th to 90th percentile band and the gray data density, over −2 to +2 SD of standardized self-efficacy; cases with all three cybervictimization items missing were excluded, and the total change was 5.7 and 9.1 percentage points.

Three auxiliary variables appear in Figure 1 and Figure 2 and are defined here. Traditional victimization is the mean of the remaining bullying items in the same TIMSS block (eight at Grade 4, eleven at Grade 8), reverse-scored so that higher values indicate more frequent victimization. School belonging is the mean of the seven (Grade 4) or eight (Grade 8) belonging items, reverse-scored. The endorsement index is the standardized person mean across the environmental-attitude block, which shares the agreement response format with the self-efficacy items but not their content and excludes every outcome analyzed here; an extremity index is the person standard deviation across the same block.

The set of control specifications shown in Figure 2b,e comprises six models of the quadratic term, each estimated within the system with jackknife standard errors: (i) the raw cybervictimization composite; (ii) traditional victimization as an outcome; (iii) school belonging as an outcome; (iv) cybervictimization residualized on traditional victimization; (v) cybervictimization with the endorsement and extremity indices entered linearly and quadratically; and (vi) both adjustments together, which we refer to as the fully adjusted specification. All six controlled home digital access and, at Grade 8, frequency of online use. These analyses are reported to show how the estimate moved as shared variance was removed; because traditional victimization is itself strongly associated with cybervictimization, specifications (iv) and (vi) removed substantive as well as method variance and are interpreted as a bounding exercise rather than a purification.

### 3.6. Functional Form

Within each system we estimated the quadratic association between the released self-efficacy score and cybervictimization with JK2 jackknife standard errors, controlling home digital access and, at Grade 8, frequency of online use, and pooled the estimates by random-effects meta-analysis with Paule–Mandel heterogeneity variance (Paule & Mandel, 1982) and Hartung–Knapp intervals, reporting prediction intervals. We then re-estimated the quadratic by logistic regression on any-versus-no victimization, and compared linear, quadratic, and restricted cubic spline specifications (five knots at the 5th, 27.5th, 50th, 72.5th and 95th percentiles) by out-of-sample weighted deviance per unit weight in held-out halves split at the school level; “fits as well as” denotes a lower or equal held-out deviance. Reproducibility of system-level estimates was assessed by 100 repeated school-level split-half correlations with Spearman–Brown correction and a bootstrap interval.

### 3.7. Simulation

The simulation addresses one question: under what measurement conditions does a routine test for curvature mislead, and does IRT scoring correct it? To answer it, a full factorial simulation crossed endpoint severity (mild, severe), local dependence (absent, strong), number of response categories (four, six), latent quadratic coefficient (0, 0.10), sample size (1000, 5000) and score type (raw summed score, true-parameter unidimensional expected a posteriori latent score), giving 64 cells with 500 replications each. Items were generated from the same logistic graded response model used for scoring, so the scoring model used the generating parameters; where local dependence was present a testlet factor loaded on items 1–3. The outcome was continuous and standardized, so that curvature was not induced by categorizing the outcome. The simulation used independent sampling and did not model the clustered design of the applied data; its Type I error and power results therefore transfer to the clustered TIMSS analysis only approximately, a qualification we carry into the interpretation.

Because the analysis regressed a standardized outcome on a standardized observed score and its square, the estimand is not the latent quadratic coefficient: the two are on different scales. We therefore defined the target as the population projection coefficient of the standardized outcome on the standardized analysis score and its square, computed in an independent reference population of 400,000 cases for every cell and score type, and reported bias and coverage relative to that estimand. Rejection of the hypothesis of no curvature was reported separately, since under a purely linear latent process a nonzero projection coefficient is itself the measurement-induced artifact of interest. Standard errors were heteroskedasticity-robust (HC1), and binomial Monte Carlo uncertainty was reported for rejection and coverage. Latent scores were computed from the true generating item parameters and therefore represented a best-case condition rather than an achievable one. We refer to them as true-parameter unidimensional EAP scores rather than oracle scores, because in the local-dependence cells the unidimensional scorer ignores the testlet factor and is not correctly specified. Because scoring with the true generating parameters is a best case, we added a realistic scoring condition in which each replication estimated the graded response model from its own item responses using independent software (Sanchez, 2021) and then scored with those estimates. That condition crossed endpoint severity, local dependence, latent quadratic and sample size at four categories, with 200 replications per cell, and was evaluated against the same population projection estimand. Full cell-level results are in the Supplementary Materials.

## 4. Results

### 4.1. Range: The Scale Measured Poorly Among Confident Children

**Figure 3:**
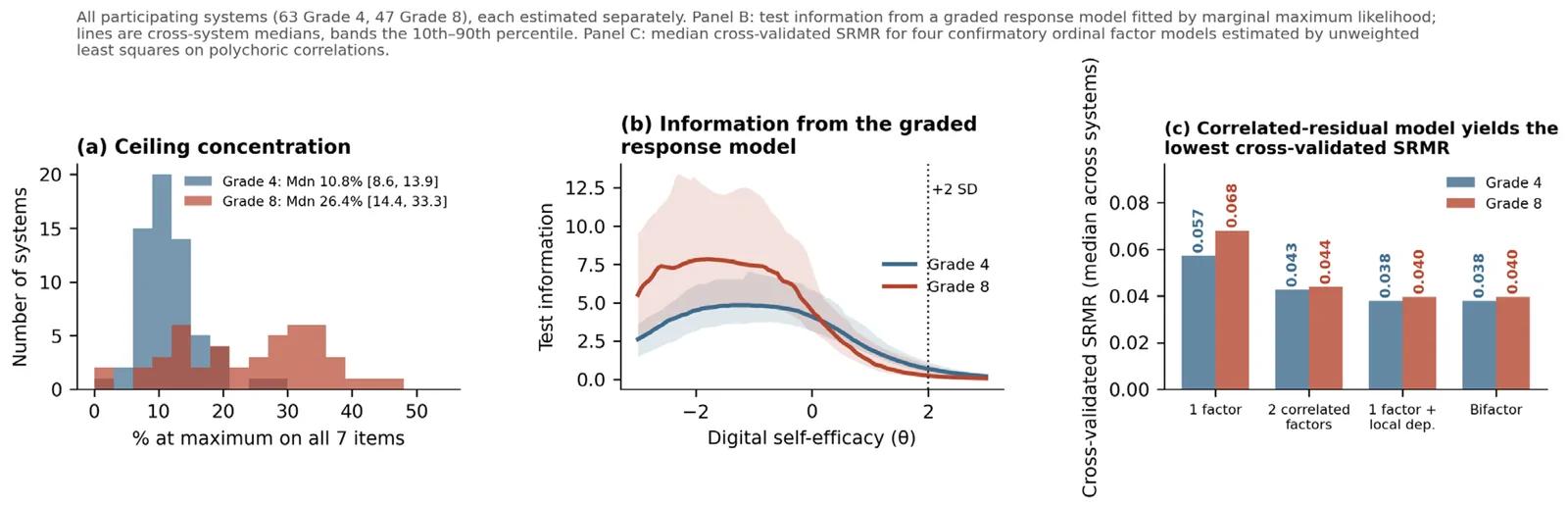
Measurement properties of the scale. (**a**) Distribution across systems of the percentage of children at the scale ceiling, with medians and interquartile ranges; (**b**) test information from graded response models fitted within each system, with lines showing cross-system medians and bands the 10th to 90th percentile; (**c**) median cross-validated SRMR for four confirmatory ordinal factor models; the correlated-residual model yields the lowest value.

At Grade 8 a median of 26.4% of children endorsed the maximum on all seven items (interquartile range 14.4 to 33.3, maximum above 45%); at Grade 4 the median was 10.8% (interquartile range 8.6 to 13.9), a mild rather than severe ceiling (Table 2; Figure 3a). Median test information fell from 4.08 at the mean to 0.68 at two standard deviations above it at Grade 4, and from 4.47 to 0.24 at Grade 8: losses of 83% and 95% (Figure 3b). Conditional standard errors at +2 SD were 1.21 and 2.06, against 0.50 and 0.47 at the mean. At +1 SD information was usable but appreciably degraded at Grade 4 (1.95, conditional standard error 0.72) and already halved at Grade 8 (1.19); the evidence concerned substantial deterioration by approximately two standard deviations above the mean rather than an immediate loss above it.

Because the standard graded response model assumes local independence, and the structure below indicated local dependence among the first three items, we confirmed the loss with a bifactor (testlet) model, computing the exact marginal information by enumerating all 4^7^ = 16,384 response patterns. The specific factor was substantial (median loading 1.01 and 1.18), and exact general-factor information fell from 3.33 to 0.66 at Grade 4 and from 3.53 to 0.33 at Grade 8 (losses of 80% and 89%); the exact and naive losses differed by a median of only 0.94 percentage points. A Grade 8 sensitivity refit with wider bounds left the loss essentially unchanged (90.8% versus 89.3%). The upper-range information loss is therefore not an artifact of the local-independence assumption. Per-sample testlet convergence and boundary diagnostics, which lead us to read the exact testlet estimates as sensitivity estimates (particularly at Grade 8), are in Supplementary Table S2.

### 4.2. Structure: Localized Covariance Rather than a Separable Dimension

A one-factor model left a median in-sample SRMR of 0.048 at Grade 4 and 0.062 at Grade 8; a correlated two-factor model gave 0.030 and 0.032, and a one-factor model with correlated residuals among items 1–3 gave 0.025 and 0.027, with the bifactor solution numerically equivalent to the latter (Table 2; Figure 3c). Because the residual-correlation and bifactor specifications are more flexible, we repeated the comparison under cross-validation. The ordering held: cross-validated SRMR was 0.057 and 0.068 for one factor, 0.043 and 0.044 for two correlated factors, and 0.038 and 0.040 for correlated residuals, with the correlated-residual specification performing at least as well as the two-factor specification in 59 of 63 and 46 of 47 systems. The estimated correlation between the two putative factors was 0.84 and 0.82. Descriptive absolute fit indices computed from the ULS discrepancy agreed with the residual comparison: median RMSEA and CFI were 0.039 and 0.988 for one factor versus 0.019 and 0.998 for correlated residuals at Grade 4, and 0.050 and 0.986 versus 0.020 and 0.998 at Grade 8 (Supplementary Materials).

We state the conclusion with the caution the evidence warrants. The results did not require a clearly separable second substantive dimension. Localized covariance among the first three items provided a plausible representation of the apparent multidimensionality, although the present covariance models could not determine whether that covariance reflected wording, task similarity, or a narrow specific factor. The correlated-residual and bifactor specifications were statistically equivalent here, so their equivalence was not independent corroboration of either interpretation. For applied purposes the practical implication is the same: the item groups should not be treated as distinct competencies, and a model allowing for their shared covariance should be preferred to one that ignores it.

Reliability supported use of the total score. Omega total was 0.83 and 0.89, omega hierarchical for the general factor 0.79 and 0.83, and omega for the item subsets 0.75 and 0.75 at Grade 4 and 0.85 and 0.83 at Grade 8. Cronbach alpha was 0.76 and 0.81 (interquartile ranges 0.74 to 0.78 and 0.78 to 0.84; full ranges 0.68 to 0.86 and 0.69 to 0.91), understating reliability as expected for ordinal items. The general factor therefore carried most of the reliable variance, and the released composite remained interpretable despite the local dependence.

### 4.3. Method Variance

The external endorsement index shared a median of 4.9% of variance with the self-efficacy score at Grade 4 (range 0.2 to 13.4) and 6.1% at Grade 8 (range 0.9 to 18.3). In the latent models, adding the common endorsement-format factor reduced the latent variance of the self-efficacy factor by 32% and 44%. These are different quantities. The external endorsement index is the primary evidence here, because it is computed in every system; the latent reduction rests on a single system and on a model that treated the four-category indicators as continuous (a categorical-indicator interaction was not feasible), so we treat it as an illustrative sensitivity estimate rather than an established quantity and give it correspondingly limited weight. Both indicate a non-trivial format component; neither identifies its psychological nature.

### 4.4. Functional Form: An Attenuated and Poorly Reproducible Conclusion

Fitted as competing theories imply, the quadratic term of digital self-efficacy predicting cybervictimization pooled to b = 0.027 [0.021, 0.033] at Grade 4 and 0.051 [0.042, 0.061] at Grade 8—by conventional standards a strongly replicated effect that might be misread as indicating an optimal level of confidence—an interpretation the remaining analyses do not support. Table 3 presents the sensitivity analyses.

Changing the outcome from the standardized composite to a binary any-versus-no indicator, holding the covariates identical, attenuated the quadratic but did not reverse it: the median coefficient was +0.009 [−0.029, 0.043] at Grade 4 and +0.026 [−0.002, 0.052] at Grade 8, positive in 40 of 63 and 33 of 45 samples. An earlier version of this analysis reported a sign reversal; that reversal was produced by the covariate set rather than by the outcome scale, because the binary model had additionally controlled traditional victimization. When traditional victimization was added as a sensitivity analysis the binary quadratic turned negative (−0.020 and −0.033), which located the reversal in the covariate adjustment and not in the choice of outcome metric.

Held-out comparison, with samples split at the school level and spline knots computed in the training half only, gave deviances whose pairwise differences were small and not statistically distinguishable from zero. The bootstrap 95% interval for the median quadratic-minus-linear held-out deviance difference was [−0.3, 0.5] × 10^−3^ at Grade 4 and [−0.6, 0.1] × 10^−3^ at Grade 8, and the spline-minus-linear interval likewise included zero ([−0.9, 0.9] and [−3.0, 0.1] × 10^−3^). Splitting the Grade 8 systems by whether knot reduction was required showed no stronger nonlinearity in the ceiling-affected subset: the spline was the best held-out specification in 11 of 21 knot-reduced and 12 of 24 full-knot systems, with comparable median advantages, so the reduced scale resolution did not conceal a larger curvilinear effect. Endpoint concentration initially produced tied training quantiles and hence non-estimable spline bases in 1 Grade 4 and 21 Grade 8 samples; we therefore selected strictly increasing knots and reduced their number when ties occurred, which yielded an estimable spline in all 63 and 45 samples, so the three specifications were compared on identical sets. A linear specification fitted at least as well as the quadratic in 34 of 63 Grade 4 and 17 of 45 Grade 8 samples. The best held-out specification was linear in 28, quadratic in 11 and spline in 24 samples at Grade 4, and spline in 23, linear in 12 and quadratic in 10 at Grade 8. No specification was clearly preferred at Grade 4; at Grade 8 the spline was most often best, but the median deviance advantage over the linear model was 0.003, which was not a material difference.

The pooled effect was heterogeneous and only moderately reproducible. Between-system heterogeneity was high (I^2^ = 77% at Grade 4 and 88% at Grade 8; τ^2^ = 4.2 × 10^−4^ and 8.3 × 10^−4^; Table 3), and every 95% prediction interval included zero, indicating that the effect in a newly sampled system was not reliably distinguishable from zero; this reflected heterogeneity and did not invalidate the pooled within-system average. Removing the five nested benchmarking systems (Abu Dhabi, Dubai and Sharjah within the United Arab Emirates; Ontario and Quebec within Canada) left the pooled quadratic essentially unchanged—0.026 [0.019, 0.032] across the 58 national Grade 4 systems and 0.051 [0.041, 0.062] across the 42 national Grade 8 systems—with every prediction interval still including zero, so the nested benchmarks did not drive the pooled result. Across 100 repeated school-level splits, the Spearman–Brown corrected split-half reliability of the system-level quadratic from the primary matched-covariate model—the composite quadratic controlling home digital access and, at Grade 8, online use—was 0.67 [0.49, 0.78] at Grade 4 and 0.73 [0.48, 0.83] at Grade 8, where each bootstrap replicate resampled samples, averaged the correlation across all splits, and only then applied the correction. Under the headline matched-covariate model, the model-implied probability of any cybervictimization changed by 5.7 percentage points at Grade 4 and 9.1 at Grade 8 between −2 and +2 SD of standardized self-efficacy, excluding cases with all three cybervictimization items missing (Figure 2c,f). Adding the common endorsement-format factor to the latent model did not attenuate the quadratic (0.028 to 0.036 and 0.021 to 0.026), so these sensitivity analyses did not support measurement error or the estimated common-format factor as sufficient explanations of the curvature.

One feature of this application merits emphasis because it qualifies the simulation. The applied models used the released TIMSS scale score, which is itself produced by an item-response model rather than by summing raw responses. The curvature reported here therefore already survived the remedy that the simulation showed to be effective against summed-score coarseness, and it could not be attributed to that coarseness. The two results are consistent because scoring and range are separate problems. A latent score removes the nonlinear compression that arises from adding ordered categories, which is what the simulation manipulated; it does not create information where the items provide none. With conditional standard errors of 1.2 at Grade 4 and 1.7 to 2.1 at Grade 8, two standard deviations above the mean, even a well-constructed latent score is imprecise in exactly the range where the exposure hypothesis applies. That is the more likely reason the applied estimate was attenuated under an alternative outcome, heterogeneous across systems, and only moderately reproducible across split halves, and it is why the range diagnostic, rather than the choice of scoring method, was the binding constraint in this application. Cross-system heterogeneity nonetheless has at least two candidate sources that this study cannot fully separate: the range/ceiling problem the workflow foregrounds, and measurement non-invariance, which ALMamari (2026) documents for this scale at Grade 8, where full scalar invariance failed, and which would by itself produce heterogeneous quadratic estimates. We emphasize the range explanation because it is what our diagnostics identify and what the simulation shows to be sufficient, but non-invariance is a credible co-contributor, and separating the two would require an invariance-constrained cross-system model beyond the present scope.

### 4.5. Simulation: When the Diagnosed Conditions Mattered

Table 4 reports the principal results; the estimator was well calibrated for its own estimand throughout (grouped bias below 0.0013, coverage 94–96%; cell-level detail in the Supplementary Materials). What changed across conditions was the estimand itself and the resulting inference.

With a purely linear latent process, endpoint concentration made the population projection coefficient on the raw summed score depart from zero: −0.0055 under mild and −0.0166 under severe concentration. Analysts testing for curvature rejected the null in 5.35% of replications under mild concentration but 16.73% under severe concentration; the Monte Carlo standard errors of these aggregated rates were 0.36 and 0.59 percentage points. True-parameter unidimensional latent scoring moved the projection coefficient to −0.0012 and −0.0004 and returned rejection to 4.75% and 5.48% (5.475% before rounding; Monte Carlo standard errors 0.34 and 0.36), indistinguishable from the nominal 5%. Because the score was computed from the true generating item parameters, and because in the local-dependence cells that scorer ignores the testlet factor, this was a best-case bound on what better scoring can achieve rather than a guarantee.

The same mechanism attenuated genuine effects. With a latent quadratic of 0.10, the projection coefficient on the raw score fell from 0.090 under mild concentration to 0.053 under severe concentration, and power fell from 89% to 71%; true-parameter latent scoring recovered the projection to 0.075 and power to 88%. Averaged over the design, the number of response categories changed null rejection by 1.25 percentage points and power by 2.65 percentage points; the largest single-condition difference was in the severe-concentration raw-score null cell, where four categories rejected in 14.35% of replications and six in 19.10%.

Scoring with estimated rather than known item parameters entailed almost no loss of accuracy. This realistic condition used 200 replications per cell rather than the 500 of the main design and covered four-category items at two sample sizes, so its rates are less precise (binomial Monte Carlo standard errors of 0.73 and 0.84 percentage points for the aggregated null rates) and should be read as supportive rather than definitive. In the comparable four-category cells, an expected a posteriori score built from parameters estimated in the analyst’s own sample rejected the null of no curvature in 4.5% of replications under mild and 6.0% under severe concentration, against 6.2% and 14.4% for the raw summed score and 4.7% and 4.5% for the true-parameter score; coverage of the projection estimand was 94.0% to 95.0%. Power at a latent quadratic of 0.10 was 91.6% and 87.5%, against 87.9% and 69.0% for the raw score, and the largest absolute departure of the mean estimate from the true-parameter estimand was 0.006. The practical recommendation therefore did not depend on knowing the item parameters: with 1000 or 5000 cases, estimating them in the same sample recovered nearly all of the benefit.

The range and structure diagnostics each detected the condition they target (endpoint mass 5.6% versus 21.2%; the local-dependence signal near zero when absent and clearly positive when present), but the two did not carry equal inferential weight: local dependence did not independently inflate false-positive curvature in this design, whereas endpoint severity did. The lesson is not that every detected problem distorts inference, but that the range diagnostic identifies one that demonstrably does.

## 5. Discussion

This article makes a methodological contribution—an integrated, ordered workflow for diagnosing short ordinal scales—and uses one newly released instrument to demonstrate it. Because readers of an applied paper need to know which conclusions are general and which belong to the specific scale, we separate the two explicitly below.

### 5.1. Methodological Contribution

The workflow is not specific to digital self-efficacy. Any short ordinal self-report scale used to study children—attitudes to school, well-being, resilience, perceived safety, attitudes to science—is exposed to the same five failures, and the same five analyses diagnose them. Three features make the procedure transferable. Its minimal diagnostic core requires only the item responses; the method-variance and functional-form stages additionally require auxiliary same-format items and the covariates, weights and cluster identifiers of the substantive analysis. Its stages are ordered so that each conditions the interpretation of the next, which prevents the common error of reporting a high alpha as though it settled dimensionality, or a significant squared term as though it settled functional form. And its outputs are quantities applied researchers can act upon—summarized as the checklist in the final column of Table 1—namely where on the latent continuum the scale is informative, whether a total score is defensible, and whether a nonlinear conclusion survives an ordinary change in specification.

The most transferable practical recommendation concerns scoring. Where endpoint concentration is substantial, an IRT-based latent score returned rejection rates to nominal levels and recovered most of the lost power in our simulation, and it did so whether the item parameters were known or estimated in the analyst’s own sample, although the estimated-parameter condition was evaluated under a narrower design (four-category items, two sample sizes, 200 replications) and should be read as supportive rather than definitive. The approach is computationally inexpensive, requires no additional data, and is available in every major software package. It is not, however, a general solution: our own applied analysis used an item-response score and still produced a curvature estimate that the remaining diagnostics found fragile, because latent scoring cannot recover information the items do not carry at the top of the range. Where a latent score is not feasible, or where the ceiling is severe enough that even a latent score is imprecise, the information function indicates where inference is supportable, and results should be confined to that region.

### 5.2. What the Application Shows About the TIMSS Digital Self-Efficacy Scale

Applied to the TIMSS 2023 Digital Self-Efficacy scale, the workflow returned a coherent but instrument-specific picture, and these conclusions should be read as properties of this scale rather than as general claims. The scale is endpoint-concentrated among adolescents and loses most of its information above the mean; its apparent second dimension is better represented as localized covariance among three similarly worded items than as a separable competency; its total score nonetheless remains interpretable, with omega hierarchical near 0.80; and it shares a non-trivial component of variance with an external same-format index, indicating some response-format influence (the larger single-system latent estimate rests on one system and a continuous-treatment model and is treated only as illustrative). The accompanying simulation established that these are not merely descriptive: endpoint concentration alone tripled the false-positive rate for curvature under a linear latent process and reduced power to detect a genuine effect, and IRT scoring substantially repaired both. Substantively, the apparently robust curvilinear association between digital confidence and cybervictimization did not survive the functional-form stage—it attenuated under a binary outcome, was not reliably present in a newly sampled system, and was only moderately reproducible—so it does not support an inference about an optimal level of confidence. This is not to say the association is an artifact: the composite-outcome quadratic remained positive and distinguishable from zero in every specification. The claim is narrower—the effect is real within systems but small, heterogeneous across systems, and specification-sensitive, so it will not reliably reproduce and cannot fix an optimal level. Where a single construct-valid representation is required, we prefer the binary any-versus-no indicator to the composite, because the three items are rare-event reports whose composite is dominated by a few frequent respondents and the binary form is closer to the question of whether a child is victimized at all; under that preferred specification the quadratic is small and often not distinguishable from zero. We report both because the divergence between them is itself informative about the fragility of the curvilinear claim.

### 5.3. Implications for Research on Children’s Digital Self-Efficacy and Cybervictimization

Perceived digital self-efficacy is not interchangeable with demonstrated competence; the scale examined here measures how capable children feel, not what they can do. This distinction matters for the questions the scale is used to answer. Endpoint concentration at Grade 8 means the scale distinguishes poorly among confident adolescents—precisely the group to which an exposure account of online harm applies—so studies that look for elevated risk at the high-confidence end are asking the scale to resolve differences it cannot measure. The three similarly worded items should not be read as a distinct competency, and reported associations should be interpreted with the shared response-format component in mind.

For research on cybervictimization specifically, the results counsel caution about threshold thinking. The pooled quadratic would, at face value, imply an optimal level of digital confidence beyond which exposure rises; but the effect was small on the probability scale—a change of roughly six to nine percentage points across the full range of confidence—heterogeneous across systems, and fragile to ordinary analytic choices, and its turning point often fell outside the region where the data are informative. Intervention designs should therefore not target a confidence “sweet spot” inferred from cross-sectional self-reports. More informative evidence would combine self-reported confidence with performance-based digital-skill assessment, direct or logged measures of online activity, and longitudinal follow-up, so that confidence, competence, exposure and harm can be separated rather than inferred from a single common-reporter instrument. A general interpretive caution underlies this. With samples of tens to hundreds of thousands of children, standardized coefficients of 0.03 to 0.05 are highly significant yet correspond to trivial changes on the scale of the outcome—here a few percentage points in the probability of any victimization across the whole range of confidence. We have therefore leaned on prediction intervals, held-out validation and probability-scale magnitudes rather than on *p*-values, and the “strongly replicated” language used earlier should be read in that light: the effects are statistically robust in the sense of recurring, but practically small.

### 5.4. Limitations and Generalizability

Several limitations qualify the measurement analyses. Factor models were estimated by unweighted least squares on polychoric matrices and compared by in-sample and cross-validated residuals; robust mean- and variance-adjusted statistics were unavailable, so we reported no χ^2^-based indices or nested difference tests. The correlated-residual and bifactor specifications were statistically equivalent in this item set, so we could not determine what the localized covariance represented. The latent estimate of endorsement-format variance came from one system, treated the four-category indicators as continuous, and could not separate acquiescence from positive self-perception or shared wording. Our invariance evidence was a loading-based approximation; for Grade 8 the definitive analysis is ALMamari (2026). Item parameters were estimated without sampling weights, which is standard but not universal practice. The substantive application was cross-sectional, with the predictor and outcome reported by the same child.

Two boundaries on generalizability deserve emphasis. First, the demonstration used international large-scale-assessment data, whose complex sampling supplies the weights, clusters and many independent systems that make the functional-form and reproducibility stages informative; outside that setting the same diagnostics apply, but the cross-system replication that here exposed the fragility of the curvilinear effect will usually be unavailable, and a single-sample study will lean more heavily on the held-out and prediction-interval components. Second, the workflow was demonstrated on a seven-item, four-category scale: the diagnostic logic transfers to other short ordinal self-reports, but the quantitative thresholds do not. How much a ceiling matters, and how much IRT scoring helps, depend on the number of items and categories and on where the population sits on the latent continuum, and our simulation spanned four to six categories and two ceiling patterns rather than the full range. Very coarse (two- or three-category) items, longer scales, or scales without a ceiling may need fewer stages or a different emphasis, and genuinely multidimensional constructs require confirmatory structural work beyond this diagnostic screen. As a modest test of transfer within the present data, we applied the range and reliability core to a second short agreement-format scale, students’ environmental attitudes: it showed the same pattern—a median upper-range information loss of 90% at Grade 4 and 82% at Grade 8, with 25% and 8% of children at the ceiling—so the diagnosed problem is not peculiar to digital self-efficacy. This second case exercised only the range and reliability components of the workflow, not the full five-stage sequence, and it comes from the same assessment program; broader applicability of the complete workflow is therefore advanced as a theoretically supported proposition rather than an empirically established conclusion, to be tested by applying all five stages to independent scales and datasets.

## 6. Conclusions

Short ordinal scales are often adopted for substantive research before their measurement properties are examined, yet nonlinear questions are routinely asked of them. The workflow set out here requires little effort, uses only the data already available, and indicates which questions the measure can answer. Applied to the TIMSS 2023 Digital Self-Efficacy scale it showed a defensible total score, a scale that measures poorly among the most confident children, and a curvilinear conclusion too fragile to support an inference about an optimal level of confidence.

## Figures and Tables

**Table 1:** An integrated diagnostic workflow for short ordinal scales.

Diagnostic Stage	Potential Failure	Recommended Analysis	Consequence If Undetected	Action If Detected
Range	Endpoint concentration	Endpoint mass; graded-response test-information and conditional-SE curves	Effects in the upper range may be compressed and imprecisely estimated	Confine inferences to the informative range, or adopt an IRT-based score before modeling
Structure	Local item dependence	Competing ordinal models (one-factor, correlated factors, correlated residuals, bifactor) with cross-validated comparison	Total-score dimensionality may be misidentified	Model the shared covariance; do not treat item subsets as separate competencies
Reliability	Specific-factor variance	Omega total, omega for each item subset, and omega hierarchical	High total reliability may conceal multidimensionality	If omega hierarchical is high, retain the total score; otherwise reconsider dimensionality before scoring
Method variance	Agreement-format covariance	External endorsement index and method-factor sensitivity model	Associations may partly reflect common response-format variance	Bound associations for shared-format variance; avoid response-style interpretations without direct evidence
Functional form	Unstable curvature	Alternative outcome specifications, splines, held-out validation, prediction intervals and practical magnitude	A quadratic term may not represent a stable substantive curve	Interpret a curve only if it survives an alternative outcome metric, a flexible form, out-of-sample validation, and a prediction interval

The final column is a practical checklist: it states the analytical decision that each diagnostic result implies. The minimal diagnostic core (range, structure, reliability) uses only the item responses; the method-variance stage additionally requires auxiliary agreement-format items, and the functional-form stage requires the outcome, covariates, sampling weights and cluster identifiers of the substantive analysis.

**Table 2:** Measurement properties of the TIMSS 2023 Digital Self-Efficacy scale across all participating systems.

Property	Grade 4 (63 Systems)	Grade 8 (47 Systems)
% at ceiling (maximum on all 7 items)	10.8 [8.6, 13.9]	26.4 [14.4, 33.3]
Test information at θ = 0 (unidimensional GRM)	4.04 [3.59, 4.64]	4.35 [3.75, 5.28]
Test information at θ = +2 SD (unidimensional GRM)	0.68 [0.57, 0.86]	0.24 [0.14, 0.54]
Information loss, θ = 0 to +2 SD (unidimensional)	83%	95%
Information loss, θ = 0 to +2 SD (bifactor/testlet)	80%	89%
Conditional SE at θ = +2 SD (unidimensional|exact testlet)	1.21|1.23	2.06|1.74
Testlet (specific-factor) loading, items 1–3	1.01	1.18
In-sample SRMR: one factor	0.048 [0.042, 0.058]	0.062 [0.057, 0.070]
In-sample SRMR: correlated two factors	0.030 [0.028, 0.038]	0.032 [0.027, 0.040]
In-sample SRMR: one factor + correlated residuals	0.025 [0.022, 0.029]	0.027 [0.022, 0.033]
Cross-validated SRMR: one factor	0.057 [0.049, 0.065]	0.068 [0.063, 0.077]
Cross-validated SRMR: correlated two factors	0.043 [0.038, 0.048]	0.044 [0.037, 0.050]
Cross-validated SRMR: one factor + correlated residuals	0.038 [0.034, 0.046]	0.040 [0.034, 0.047]
Systems where correlated residuals ≤ two factors (cross-validated)	59/63	46/47
Correlation between the two putative factors	0.838 [0.817, 0.868]	0.815 [0.781, 0.842]
Omega total	0.828 [0.812, 0.844]	0.885 [0.861, 0.910]
Omega, items 1–3	0.747 [0.711, 0.773]	0.853 [0.795, 0.876]
Omega, items 4–7	0.747 [0.722, 0.771]	0.826 [0.796, 0.861]
Omega hierarchical (general factor)	0.787 [0.770, 0.805]	0.833 [0.798, 0.866]
Cronbach alpha	0.756 [0.736, 0.775]	0.811 [0.779, 0.838]
Variance shared with external endorsement index	4.9% [0.2, 13.4]	6.1% [0.9, 18.3]
Latent variance reduction, endorsement-format factor (illustrative, one-system sensitivity estimate)	32%	44%

Entries are cross-system medians with interquartile ranges in brackets. Graded response models were fitted by marginal maximum likelihood within each system; the bifactor (testlet) model allows a specific factor on items 1–3 and its information refers to the general factor. Factor models were estimated from polychoric correlations by unweighted least squares; cross-validated SRMR evaluates parameters estimated on one random half of each system against the polychoric matrix of the held-out half. The external endorsement index is built from the environmental-attitude block and is not a bound in either direction. The final row is an illustrative, one-system sensitivity estimate from a model that treated the four-category indicators as continuous, and should be read with corresponding caution; the primary evidence for a response-format component is the external endorsement index (row above), available in every system.

**Table 3:** Sensitivity of the cybervictimization analysis to measurement and modeling choices.

Diagnostic	Grade 4	Grade 8
Quadratic on standardized composite (pooled)	+0.027 [0.021, 0.033]	+0.051 [0.042, 0.061]
Pooled quadratic, national systems only (nested benchmarks removed)	+0.026 [0.019, 0.032] (k = 58)	+0.051 [0.041, 0.062] (k = 42)
Quadratic on composite, matched covariates (median)	+0.0281	+0.0558
Quadratic on binary outcome, matched covariates (median [IQR])	0.0093 [−0.0291, 0.0434]	0.0259 [−0.0020, 0.0523]
Systems with positive binary quadratic	40/63	33/45
Sensitivity: binary quadratic additionally controlling traditional victimization	−0.0203	−0.0328
95% prediction interval, fully adjusted	[−0.002, 0.039]	[−0.015, 0.055]
Between-system heterogeneity of the pooled quadratic (I^2^, τ^2^)	I^2^ = 77.1%, τ^2^ = 0.00042	I^2^ = 88.4%, τ^2^ = 0.00083
Held-out deviance: linear|quadratic|spline	1.1802|1.1779|1.1776	1.3197|1.3191|1.3166
Spline estimable (systems)	63/63	45/45
Best held-out specification (linear/quadratic/spline)	28/11/24	12/10/23
Linear fit at least as well as quadratic	34/63	17/45
Split-half reliability, primary model (Spearman–Brown [95% CI])	0.665 [0.487, 0.775]	0.725 [0.478, 0.834]
Latent quadratic without|with endorsement-format factor	0.028|0.036	0.021|0.026

Pooled estimates are from random-effects meta-analysis with Paule–Mandel heterogeneity variance and Hartung–Knapp intervals. Rows 2–4 use identical covariates (home digital access, and at Grade 8 online use) across the composite and binary specifications, so only the outcome metric differs; row 5 adds traditional victimization as a sensitivity analysis. Held-out deviance is weighted deviance per unit weight in halves split at the school level, with spline knots computed in the training half only; lower values indicate better out-of-sample fit. Split-half reliability is the Spearman–Brown correction of the mean correlation across 100 school-level splits, with a bootstrap interval in which samples are resampled and the correction applied within each replicate. Latent quadratic rows are STDYX standardized coefficients.

**Table 4:** Simulation: the projection estimand, rejection of no curvature, and the value of latent scoring.

Quantity	Raw Summed Score	Latent (EAP) Score
Population projection coefficient, latent b_2_ = 0	−0.0055 (mild), −0.0166 (severe)	−0.0012 (mild), −0.0004 (severe)
Rejection of no curvature, latent b_2_ = 0, mild	5.35% (MCSE 0.36)	4.75% (MCSE 0.34)
Rejection of no curvature, latent b_2_ = 0, severe	16.73% (MCSE 0.59)	5.47% (MCSE 0.36)
Grouped mean bias vs. projection estimand (max across displayed conditions)	≤0.0013	≤0.0013
Cell-level absolute bias (max)/coverage range [pooled across score types]	0.00446/92.4–98.2%	—
Population projection coefficient, latent b_2_ = 0.10, severe	+0.0535	+0.0747
Power, latent b_2_ = 0.10, mild	89.1%	92.8%
Power, latent b_2_ = 0.10, severe	70.8%	88.5%
Estimated-parameter EAP: rejection, latent b_2_ = 0 (mild|severe)	—	4.5%|6.0% (MCSE 0.73, 0.84)
Estimated-parameter EAP: power at b_2_ = 0.10 (mild|severe)	—	91.6%|87.5%
Local dependence: rejection under the null (absent|present) [pooled across score types]	8.4%|7.8%	—
Local dependence: power at b_2_ = 0.10 (absent|present) [pooled across score types]	86.6%|83.9%	—
Workflow diagnostic: endpoint mass	5.6% (mild) vs. 21.2% (severe)	—

Full factorial design crossing endpoint severity, local dependence, number of response categories, latent quadratic coefficient, sample size and score type; 64 cells with 500 replications each. Entries are averaged over the remaining factors. The target parameter is the population projection coefficient of the standardized outcome on the standardized analysis score and its square, estimated in an independent reference population of 400,000 cases per cell; bias and coverage are relative to that estimand. Standard errors are heteroskedasticity-robust (HC1); SE in parentheses is the binomial Monte Carlo standard error. Latent scores used the true generating item parameters and were therefore a best-case, not an achievable, condition; in the local-dependence cells the unidimensional scorer additionally ignores the testlet factor. Mean Monte Carlo standard error for the quadratic was 0.0009. The estimated-parameter rows come from a separate realistic condition in which each replication estimated the item parameters from its own responses using independent software and then scored with those estimates; it used four-category items and 200 replications per cell, and in those same cells the raw score rejected the null in 6.2% (mild) and 14.4% (severe) of replications, with power 87.9% and 69.0%. Its aggregate rejection rates were computed over 800 replications per ceiling level (four cells × 200 replications), giving binomial Monte Carlo standard errors of 0.73 and 0.84 percentage points. The local-dependence rows are pooled across both score types, as marked in the row labels. Cell-level results, and a worked example showing how one displayed condition aggregates the underlying design cells, are in the Supplementary Materials.

## Data Availability

The TIMSS 2023 international public-use database is available from the IEA (Grade 4: https://doi.org/10.58150/IEA_TIMSS_2023_G4_data_edition_1, accessed on 18 August 2026; Grade 8: https://doi.org/10.58150/IEA_TIMSS_2023_G8_data_edition_1, accessed on 18 August 2026). All analysis code, the per-system estimates and diagnostics (Supplementary Tables S1 and S2), cell-level simulation summaries, and the Mplus input and output files are provided as Supplementary Materials; the supplied scripts regenerate the analytic files from the public-use data. The prepared Mplus .dat files are not included and can be regenerated with the supplied make_dat.py script.

## Supplementary Materials

The following supporting information can be downloaded at: https://www.mdpi.com/article/10.3390/bs16091485/s1, Data: Table S1, per-sample estimates for all 110 samples; Table S2, convergence diagnostics and exact testlet information; item-level testlet parameters with per-parameter boundary flags; the Grade 8 sensitivity refit; the independent-software validation against the girth package; the stricter-convergence check; the subsample adequacy check; Cronbach alpha for every system; cell-level output for the main simulation and for the realistic scoring condition with estimated item parameters; item-response, factor-model, meta-analytic, control-specification, multilevel, held-out and split-half results, with the saved split matrices. Code: every analysis script. Mplus: input and output files for the latent models with the variable-order lists. The archive includes a README documenting each file and the steps required to reproduce every analysis; random seeds are fixed throughout, so the simulation and resampling results reproduce exactly.
